# Editorial: Unraveling enzyme-substrate dynamics in protein acylation and disease implications

**DOI:** 10.3389/fmolb.2026.1842734

**Published:** 2026-04-14

**Authors:** Shuyuan Cheng, Tong Liu, Jing Xing, Guoliang Zhang, Lin Zhang, Wei Zhao

**Affiliations:** 1 Department of Biomedical Engineering, Southern University of Science and Technology, Shenzhen, China; 2 Faculty of Synthetic Biology, Shenzhen University of Advanced Technology, Shenzhen, China; 3 State Key Laboratory of Quantitative Synthetic Biology, Shenzhen Institute of Synthetic Biology, Shenzhen Institutes of Advanced Technology, Chinese Academy of Sciences, Shenzhen, China; 4 State Key Laboratory for Conservation and Utilization of Bio-Resources in Yunnan, Yunnan University, Kunming, China; 5 Lingang Laboratory, Shanghai, China; 6 National Clinical Research Center for Infectious Diseases, Guangdong Key Laboratory for Diagnosis and Treatment of Emerging Infectious Diseases, Shenzhen Key Laboratory for Infectious Diseases, Shenzhen Third People’s Hospital, Shenzhen, China; 7 Institute of Emerging Agricultural Technology, Shenzhen University of Advanced Technology, Shenzhen, China; 8 Department of Food Science and Technology, Shanghai Jiao Tong University, Shanghai, China

**Keywords:** crotonylation, disease biomarkers, histone acetylation, post-translational modifications, therapeutic targets, ubiquitination

## Introduction

Protein post-translational modifications (PTMs), including acylation, ubiquitination, and other modifications, are the key regulators of gene expression and cellular homeostasis. Dysregulation of these modification dynamics is closely linked to the pathogenesis of diverse human diseases, from cancer to inflammatory disorders. This Research Topic aims to assemble cutting-edge studies that decode the molecular mechanisms of PTMs and their implications in clinical potentials, providing new insights for disease diagnosis, prognosis, and targeted therapy. Below, we synthesize the core findings of four contributing articles, contextualize their scientific significance, and highlight the collective advances of this Research Topic.

### Crotonylation regulation of cancer chemoresistance

A central focus of this Research Topic is the role of epigenetic modifications in cancer progression and therapeutic resistance. Sun et al. uncovered a novel mechanism by which p53 deficiency induces cisplatin resistance in colorectal cancer. Their study identified that p53 deficiency downregulates the decrotonylase SIRT7, leading to enhanced crotonylation of ribonucleotide reductase subunit M2 (RRM2) at the K283 site. This modification promotes RRM2 expression and activity, upregulates cisplatin resistance-related genes (ATP7A, ATP7B, MUC16) and inhibits apoptotic signaling (cleaved-PARP1/cleaved-caspase3), ultimately facilitating cancer cell survival under chemotherapy. This contributing work not only reveals a new epigenetic regulatory pathway in cancer but also identifies RRM2 crotonylation as a potential therapeutic target to overcome chemoresistance, addressing an unmet clinical need in colorectal cancer treatment.

### Epigenetic biomarkers for sepsis diagnosis and prognosis

Another critical theme of this Research Topic is the identification of epigenetic-related biomarkers for disease diagnosis. Cheng et al. focused on sepsis, a life-threatening inflammatory disorder characterized by immune dysregulation. They utilized integrated bioinformatics approaches including transcriptomic analysis and machine learning to screen histone acetylation-related biomarkers. Several key biomarkers, such as BLOC1S1, NDUFA1, and SFT2D1, were identified as a predictive nomogram and showing strong diagnostic potential. Furthermore, single-cell RNA sequencing linked these biomarkers to macrophage activity, shedding new light on the immunological mechanisms of sepsis. This contributing work provides a panel of epigenetic biomarkers for early sepsis diagnosis and risk stratification, which could significantly improve patient outcomes.

### Ubiquitination in the host-pathogen interplay

The contributing work by Feng et al. expands our understanding of the host-pathogen interplay during *Mycobacterium tuberculosis* (Mtb) infection. Through a systematic analysis of the protein ubiquitinome in human macrophages, the authors identified over 1,600 proteins with altered ubiquitination levels upon Mtb infection. Notably, the study revealed that these ubiquitination changes extensively affect not only immune-related pathways like autophagy and NF-κB signaling, but also fundamental cellular processes such as ribosome biogenesis, spliceosome, and mRNA surveillance. This comprehensive resource sheds light on how microbes manipulate host post-translational modifications to survive, providing critical insights into the molecular basis of infectious diseases and potential therapeutic targets.

### Lactylation in regulating metabolism and disease

Zhao et al. provided a comprehensive review of lysine lactylation, a relatively newly identified epigenetic modification. They summarized current knowledge regarding its discovery history, regulatory enzymes, and detection strategies. Notably, the review clarifies that lactylation encompasses three isomers—K^L-la^, K^D-la^, and K^ce^—and emphasizes that its regulatory mechanisms exhibit strong evolutionary conservation across both prokaryotes and eukaryotes. As a critical ‘metabolic-epigenetic bridge’, lactylation links cellular metabolic status to gene regulation in diverse diseases, providing a theoretical foundation for targeting metabolic-epigenetic crosstalk in future treatments.

## Conclusion

Collectively, the studies in this Research Topic underscore a central theme, i.e., the intricate interplay between protein post-translational modifications and human disease regulations. A key future direction will be to move beyond individual modifications and explore the complex crosstalk between these diverse PTMs. Understanding their collective impact on disease pathogenesis will be essential for developing the next-generation of combination therapies that target multiple epigenetic and metabolic nodes. We thank all contributing authors for their valuable work, which has made this Research Topic a comprehensive resource for researchers in biosciences and clinical medicine. We also appreciate the efforts of reviewers in shaping this collection.

